# Strengths, gaps, and future directions on the landscape of ethics-related research for spinal cord injury

**DOI:** 10.1038/s41393-023-00897-z

**Published:** 2023-04-18

**Authors:** Anna Nuechterlein, Lydia Feng, Alaa Yehia, Judy Illes

**Affiliations:** Neuroethics Canada, Division of Neurology, Department of Medicine, University of British Columbia, 2211 Wesbrook Mall, Koerner S124, Vancouver, BC V6T 2B5 Canada

**Keywords:** Public health, Outcomes research

## Abstract

Spinal cord injury (SCI) affects between 250,000–500,000 people globally each year. While the medical aspects of SCI have received considerable attention in the academic literature, discourse pertaining to its ethical implications is more limited. The experience of SCI is shaped by intersecting demographic and identity factors such as gender, race, and culture that necessitate an intersectional and value-based approach to ethics-related research that is properly situated in context. Given this background, we conducted a content analysis of academic studies exploring the perspectives and priorities of individuals with SCI published in peer-reviewed journals in the decade between 2012–2021. Terms pertaining to SCI and ethics were combined in a search of two major publication databases. We documented overall publication patterns, recruitment and research methods, reporting of demographic variables, and ethics-related discourse. Seventy (70) papers met inclusion criteria and were categorized by their major foci. Findings reveal a gap in reporting of participant demographics, particularly with respect to race and ethnicity, geographic background, and household income. We discuss these person-centered themes and gaps that must be closed in the reporting and supporting of SCI research.

## Introduction

Spinal cord injury (SCI) affects between 250,000–500,000 people each year globally (https://www.who.int/news-room/fact-sheets/detail/spinal-cord-injury). Adverse health outcomes associated with SCI include depression [1] and secondary health conditions [2] such as bladder dysfunction, neuropathic pain, pressure sores, and spasticity. While the medical aspects of SCI have received considerable attention in the literature, a smaller body of work has explored ethical issues pertaining to treatment and research in SCI [3, 4], such as informed consent, clinical decision-making, and the patient-physician relationship.

The perspectives, priorities, and experiences of people living with SCI are shaped by social, environmental, clinical, and injury-related factors. The sudden-onset and life-altering nature of traumatic SCI may lead to experiences of vulnerability and powerlessness, particularly during the early phases of injury [5]. Paternalistic approaches to care and power imbalances between people with SCI and healthcare professionals, caregivers, and other members of society during rehabilitation and community integration can contribute to experiences of vulnerability and hinder decision-making that aligns with values [6]. The experience of living with SCI is further compounded by issues of justice and fairness at a systems level, such as a lack of accessible facilities, information, and specialized rehabilitation and healthcare services that create barriers to reintegration [7] and differentially impact people living in developing countries and low-resourced and rural communities [8, 9] Sociocultural factors such as discrimination further impact adjustment after injury, with marginalized and racialized groups most affected [10].

Here we applied an intersectional and value-based approach to explore trends and themes in peer-reviewed publications devoted to understanding different aspects of the perspectives and experiences of individuals with SCI, and the ethics-related variables that shape them. We discuss critical person-centered ethics themes in this body of literature, and gaps that must be closed in the reporting and supporting of SCI research.

## Materials and methods

### Search strategy

Drawing upon methods by Tricco et al. [11], we carried out an extensive search of academic papers reporting on empirical studies published in peer-reviewed journals between 2012–2021. We searched Google Scholar and PubMed databases with two primary key terms: (1) spinal cord injury and (2) ethics. The terms {spinal cord injury}, {spinal cord injury repair}, {paraplegia}, {tetraplegia}, {quadriplegia} were combined with {ethic}, {autonomy}, {patient values}, {patient priorities}, {patient preferences}, {patient experiences}, {decision-making}, {quality of life}, {coping}, {adjustment}, {acceptance}, and {resilience} using all permutations of these terms. Titles and abstracts were screened for inclusion by the first author (AN). Inclusion criteria were accessible, English-language full-text papers on studies with the primary purpose of investigating qualitative features of the life of adults (≥18 years of age) with either traumatic or non-traumatic spinal cord injury. Exclusion criteria were: primary focus on efficacy of specific interventions; secondary health conditions and/or comorbid psychological outcomes; physical, functional, environmental, or occupational barriers; and the testing of a model or theory. Opinion papers, editorials, letters to the editor, conference abstracts or proceedings, and other grey literature were not included in this review. Systematic, scoping, integrative, and realist review designs were mined for relevant articles for inclusion, but themselves excluded. Final decisions about inclusion were determined by two independent reviewers (AN and LF). Disagreements between the reviewers any each stage of the selection process were resolved through discussion. JI undertook final decision-making where consensus could not be reached.

### Data extraction and content analysis

All authors contributed to data extraction. We collected and examined data pertaining to year of publication, country of affiliation of corresponding authors, journal, research foci, design, and recruitment methods, participant demographics, ethics discourse, and the content of limitations. We used a content analysis strategy [12] to identify ethics discourse. To this aim, we identified a priori ethics concepts from the literature on biomedical ethics, clinical ethics, and disability ethics. Concepts were refined iteratively to account for emergent ethics content. A rich coding strategy allowed content to be assigned to multiple ethics categories (e.g., justice, access) when applicable. All papers retrieved were imported to the qualitative data analysis software NVivo QSR 12 and coded by the first author (AN). To achieve intercoder reliability, AY independently coded a randomly selected sample of 20% of the publications. As for inclusion, codes were reviewed together and discrepancies discussed; if agreement could not be achieved, JI made the final call. After coding the initial sample, AY independently coded an additional 10% of the publications. Codes were compared and percent agreement was achieved at 96%.

## Results

### Overarching features of the articles

Seventy (70) papers met inclusion criteria of a total initial return of 336. The majority (*N* = 47) used qualitative study designs exclusively; 19 used quantitative methods, and 4 used mixed methods. Articles were associated with corresponding authors from institutions from 21 different countries, with the majority from the United States (*N* = 17), Canada (*N* = 11), and Australia (*N* = 7). Papers were derived from 32 different journals, and the majority of journal types represented the specialized disciplines of spinal cord injury research (*N* = 25), rehabilitation (*N* = 11), and psychology (*N* = 8).

### Major foci

**Fig. 1:**
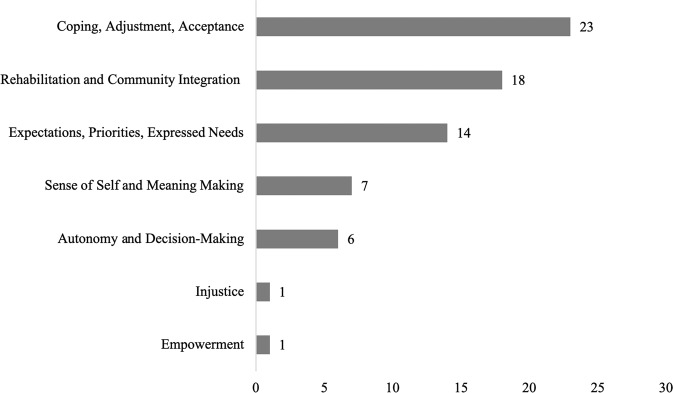
Number of total articles (*N* = 70) by major focus. *Other: Injustice, Empowerment.

Publications were categorized according to the major foci identified (Fig. 1). Coping, Adjustment, Acceptance (*N* = 23): psychosocial factors that influence the experience of SCI and overcoming adversity; Rehabilitation and Community Integration (*N* = 18): experiences of individuals with SCI during rehabilitation and in the transition back into the community; Expectations, Priorities, Expressed Needs (*N* = 14): perspectives of individuals with SCI about resources such as healthcare services and expectations and priorities regarding care and recovery; Sense of Self and Meaning-Making (*N* = 7): continuity or disruption of identity and assigning meaning to a spinal cord injury; Autonomy and Decision-Making (*N* = 6): individual decision-making, values, and priorities. Two (2) papers were not categorized due to their unique foci on Injustice (*N* = 1) and Empowerment (*N* = 1), respectively.

### Research design and recruitment methods

The majority of studies (*N* = 62) used cross-sectional research designs. The most common methods of data collection were through interviews (*N* = 45) and surveys (*N* = 22). Participants were recruited through inpatient recruitment (21/70; 30% of studies), mailed invitations (15/70; 21%), non-profit patient databases (15/70; 21%), clinical patient databases (12/70; 17%), and recontact from previous studies (9/70; 13%).

### Reporting of participant demographics

**Fig. 2:**
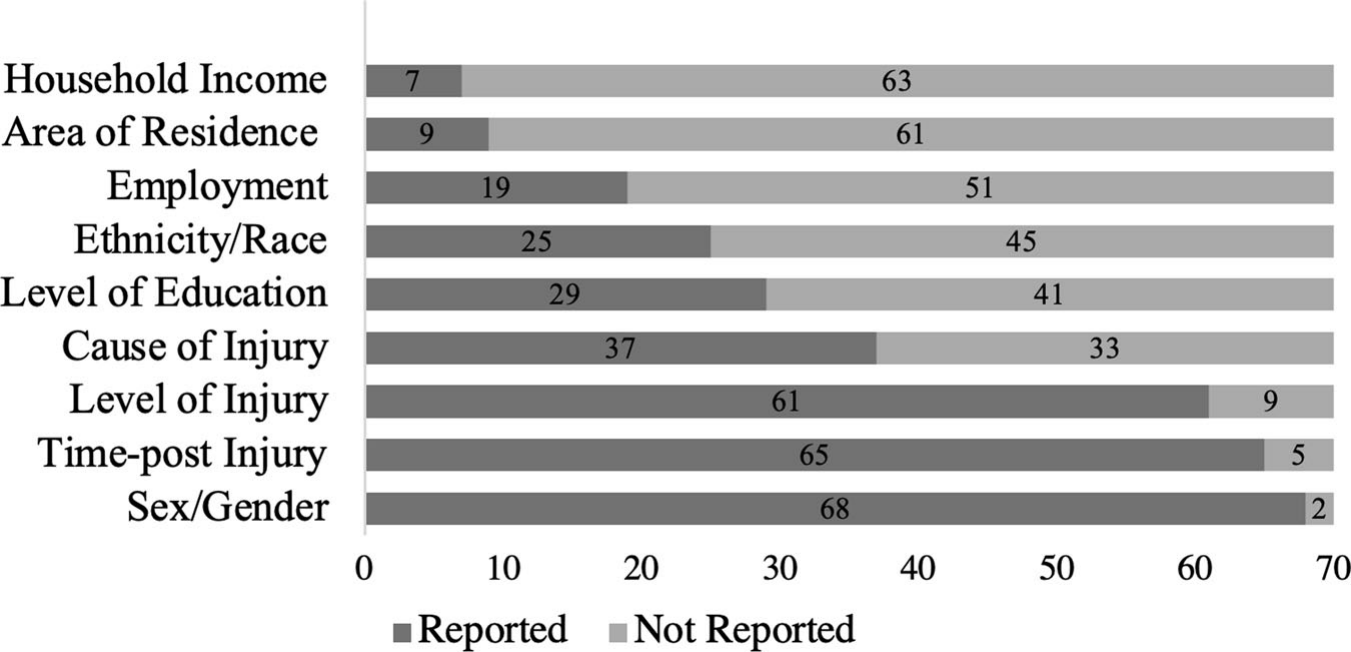
Participant demographic variables reported in studies (*N* = 70).

Sex and gender were the most frequently reported variables (68/70; 97%). More studies reported male and female as gender (38/68; 56%) than sex (16/68; 24%). Information pertaining to participant race or ethnicity was not consistently reported (25/70; 36% of studies). Reporting on household income (7/70; 10%) and area of residence (9/70; 13%) was the most limited (Fig. 2).

### Ethics discourse

**Fig. 3:**
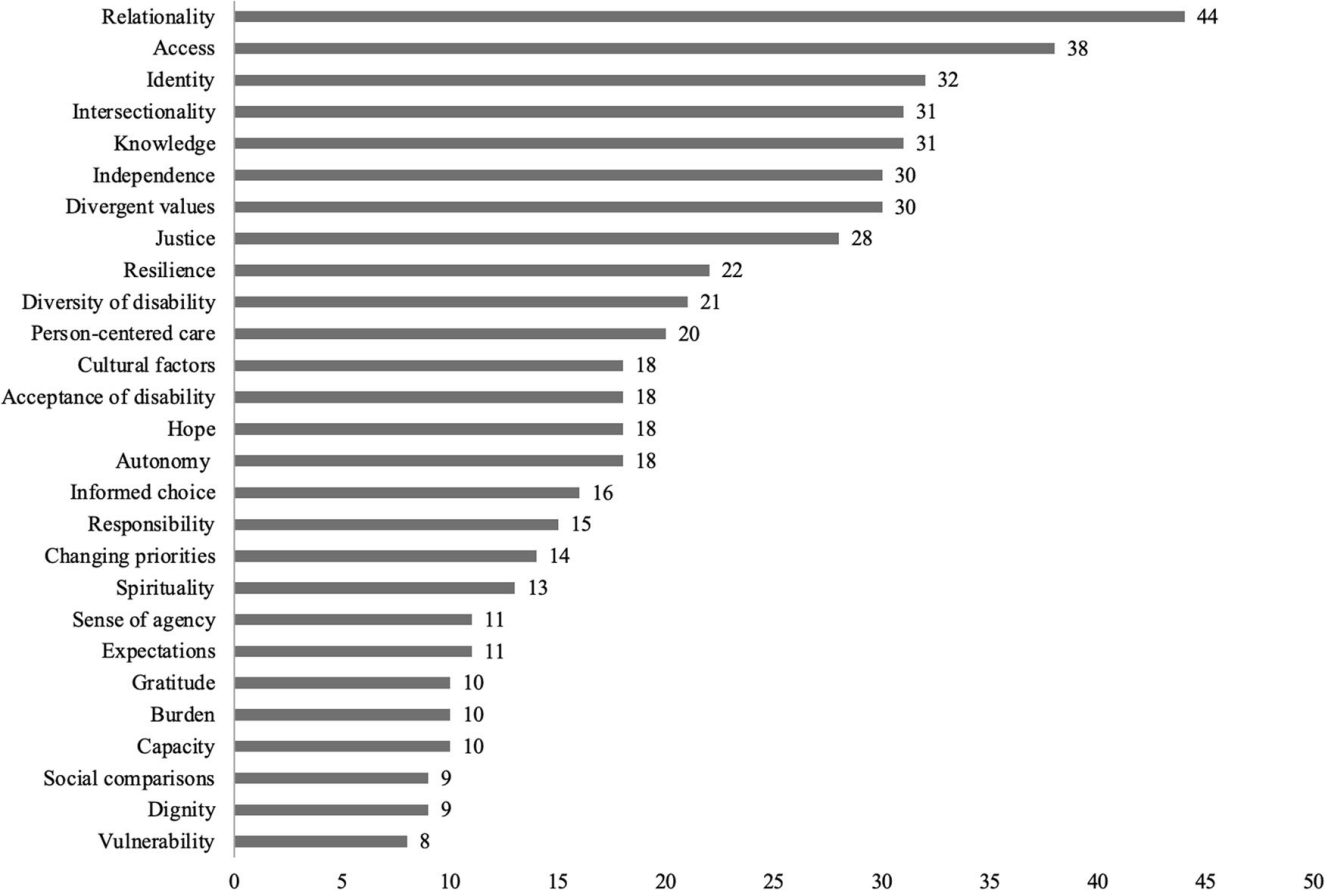
Ethics-related concepts identified by number of articles in which they are referenced.

We identified 27 distinctive ethics-related concepts in this body of literature (Fig. 3). We describe the eight coded most frequently in detail here.

### Relationality

The role of family members, peers, healthcare providers, and others close to the lives of individuals with SCI was the most frequently discussed ethics-related theme (62%; 44/70), referenced in the majority of publications that focused on Autonomy and Decision-Making (5/6), Sense of Self and Meaning Making (6/7), Coping, Adjustment, Acceptance (15/23), and Expectations, Priorities, Expressed Needs (11/18). Discourse pertaining to relationships was largely positive in nature: for example, supportive attitudes and respect from healthcare providers can facilitate goal-setting and instill hope, and peers with lived experience are viewed as an invaluable source of social support as they offer critical insights into managing the physical and psychosocial complexities of SCI. However, fear of changing relationships and burdening family members and caregivers was also discussed (14%; 10/70).

“Participants expressed the importance of a peer mentor/supporter throughout the process of dealing with the new self and of finding ways to minimize barriers to involvement. They expressed that peer mentors provided a cultural perspective of what it is to heal and recover within the local context” [13].

### Access

Access encompasses the social and environmental conditions that promote or prevent individuals with SCI from accessing or using knowledge and resources, including appropriate healthcare and rehabilitation services. This theme was discussed in 54% of the publications (38/70), particularly in those focused on Expectations, Priorities, Expressed Needs (11/14) and Rehabilitation and Community Integration (11/18). Access issues were pronounced in studies involving participants with SCI living in rural, remote, or developing regions, including communities in Western Canada and low-resourced countries, such as South Africa, Sri Lanka, Nepal, and Colombia. A few studies noted that individuals living in rural or remote communities preferred and valued their location, despite limited access.

“Accessibility to health care services could be limited by environmental and distance barriers, particularly for those in rural areas. Long wait times to access both primary care and specialist services, however, were very common which jeopardized health and well-being” [8].

### Identity and life purpose

There was substantive discussion devoted to changes or reconciliation in identity and life purpose experienced by people with SCI (46%; 37/70). All papers with a major focus on Sense of Self and Meaning Making (7/7) and 61% of publications on Rehabilitation and Community Integration (11/18) discussed this concept. Several of the articles described the life-altering nature of traumatic SCI and its impact on individuals’ perceived social roles within society, which may be culturally influenced. Others considered how sense of self is unchanged or even strengthened after injury.

“Most participants expressed that they became ‘completely different’ and ‘actually better’ people although their ‘core character still remains’” [14].

### Intersectionality

Discourse on intersectionality (44%; 31/70) relates to how the experience of SCI is shaped by intersecting social and political identity factors, including gender, race, and socio-economic status, which can add to a person’s relation to power and vulnerability. The majority of publications in the category of Expectations, Priorities, Expressed Needs discussed this theme (10/14). Most discussion of intersectionality specifically mentioned or elaborated upon differences in perspectives and experiences on the basis of gender or sex (74%; 23/31), for example in the context of resilience, perceived injustice, and risk perception.

“Women with SCI, like all women, are aware of their physical vulnerability as compared to men. It is logical to expect this sense of physical vulnerability to be as great or even greater for woman with SCI” [15].

### Knowledge

Knowledge refers to the ways in which information and education on living with disability influences the experience of SCI (44%; 31/70), and was coded in the majority of papers on Expectations, Priorities, Expressed Needs (10/14), Rehabilitation and Community Integration (12/18), and Autonomy and Decision-Making (4/6). Seeking information on management of secondary health conditions and physical limitations was discussed as a facilitator to decision-making, adjustment, and community reintegration. However, individuals reported frustration when information received in healthcare and rehabilitation facilities was not delivered in a sensitive manner or did not extrapolate to living in the broader community. Peers were often cited as an influential source of knowledge and learning, particularly during the early phases of recovery. Health literacy among older adults with SCI was explored in-depth in one article [16].

“Most of the informants stressed the need to take responsibility for their care and rehabilitation, but that to do so required that they were well informed. They wanted to know as much as possible about their current situation and prognosis, even when the information or prognosis was negative” [17].

### Divergent values and priorities

The diverse and potentially conflicting views and values between individuals with SCI and rehabilitation professionals, physicians, caregivers, family members, and or other members of society (43%; 30/70) was another prevalent ethics theme, and was discussed in all papers focused on Autonomy and Decision-Making (6/6) and the majority in Expectations, Priorities, Expressed Needs (8/14) and Sense of Self and Meaning Making (4/7). Most (63%; 19/30) specifically discussed divergent views between patients with SCI and healthcare providers; for example, perceived lack of empathy and understanding by physicians, and paternalistic approaches to care and rehabilitation. The desire to “be heard” was an overarching theme in participants’ descriptions of their relationship with some heath care providers.

“Some patients felt they ‘had no choice’ and ‘had to accept’ […] the doctor’s decision. Most of the time they were not aware that there are other options available, and felt that the decision was top–down instruction” [18].

### Independence

Independence refers to the ability to fulfill aspects of daily living and self-management without external aid (43%; 30/70). Discussion of this theme did not comprise the majority of any research category, but lack of accessible environments and reliance on others for personal activities were discussed as points of frustration for people with SCI. Access to resources, education, and supportive attitudes from family members and healthcare providers ameliorated perceptions of independence. While most papers contextualized independence as a goal for rehabilitation and community integration, a few raised concerns about the term. For example, we found criticism of the Western-individualistic view that equates functional independence with autonomy, discussions of how pursuits for physical independence may lead to further health risks, and explorations of negative external pressures toward achieving independence in the context of parenting. Independence was more frequently discussed than autonomy (18/70; 26%), and we note that, in some cases, these terms were used interchangeably.

“Taking responsibility for oneself was experienced as an intense desire for independence. Independence meant freedom, to not having to rely on others. An example mentioned by several participants was the first occasion when they were able independently to lift the wheelchair into their car and drive away […]” [19].

### Justice

Justice relates to the principle that individuals should receive what they are morally due as human beings in society, and references to it were distributed across the research categories. Discourse pertaining to justice mainly focused on social acceptance and inclusion, discrimination, stigmatization, and equal opportunities and rights for individuals with SCI and other disabilities (39%; 27/70):

“Participants stated that they experience discrimination and exclusion because of the disregard by society and authorities of the issues facing people with SCI, and the disrespect for and negligence of people with SCI” [20].

One paper specifically focused on perceived injustice among individuals with SCI. The authors concluded that perceptions of injustice may be attributed to a lack of understanding from others, rather than feelings of fault or blame about the cause injury [21].

## Discussion

We located and analyzed a sample of 70 papers published between 2012–2021 that report on research pertaining to perspectives, priorities, and experiences of individuals with SCI. Papers represent scholarship from primary authors located in 21 different countries, with the majority from highly-resourced countries situated in Western cultural contexts. The major foci of the publications reflect experiences with coping and adjustment, rehabilitation and community integration, and the expressed needs and priorities of persons affected by SCI. A smaller selection of papers focussed primarily on selfhood, autonomy, and decision-making. Discussion of acute issues—such as those pertaining to initial surgical interventions or medications—was limited.

Reporting of participant demographic variables was, overall, a point of weakness within this sample of publications. We found that in over half of the studies, variables pertaining to sex (male/female) were conflated with gender, where sex is a biological phenomenon and gender is a construct influenced by social, cultural, and behavioural factors (http://www.cihr-irsc.gc.ca/e/49347.html). Moreover, we found that demographic variables pertaining to household income, area of residence, and employment were reported in fewer than one-third of the publications, and education and race or ethnicity in less than half, similar to previous findings [22]. Level of education, location of residence, occupation, and race are social determinants of health that can contribute to marginalization [23], and racial disparities have been shown to affect health outcomes in SCI [24]. Increased transparency and accuracy in the reporting of participant demographic variables that relate to health outcomes is imperative to advance ethics scholarship in SCI through an intersectional lens.

We identified a diverse range of ethics-related discourse in this body of literature. Over 60% of papers discussed the nature and implications of relationships and social support for individuals with SCI. Indeed, perceived social support is a key determinant of life satisfaction among individuals with SCI [25], and relationships with family and friends has been identified as one of the highest life priorities in this population [26]. Reflecting the importance of relationality is an extensive body of literature devoted to understanding the critical role of family and caregivers [27] and, increasingly, peer mentorship [28] in the context of SCI.

We also found a common thread of discourse pertaining to divergent values and priorities, specifically with regard to perceptions of individuals with SCI toward health care providers. Paternalism in healthcare settings and conflicting views within the patient-physician relationship has been explored in the literature [29, 30], motivating a movement toward person-centred care and shared decision-making approaches. Indeed, engaging patients in healthcare management and decision-making is associated with improved health outcomes [31]. Empirical work to identify recovery priorities for individuals with SCI [32] has revealed that the priorities of researchers do not necessarily align with those of affected individuals, motivating changes in research foci and aims. Understanding the nature of divergent values and priorities in the context of healthcare may inform physician behaviour to better meet the needs of the SCI community. More studies devoted to disentangling the specific nature of differing values and priorities in rehabilitation, healthcare, and community settings as well as the perspectives of healthcare professionals in the context of SCI would enrich this body of literature.

Intersectionality was discussed in nearly half of the papers, particularly with respect to sex and gender differences, despite errors we note above in regards to conflation of these terms. Discourse pertaining to intersectionality reflects an appreciation that the values and experiences of people with SCI are mediated by unique and intersecting identity factors. Differences in health outcomes and experiences on the basis of sex and gender in SCI have been explored in the literature, for example, in the context of community integration [33]. Future scholarship would be strengthened with improved reporting and transparency of demographic variables, and the prioritization of efforts to include individuals with SCI from diverse and marginalized backgrounds in research. Indeed, over 25% of publications acknowledged the homogeneity of the pool of participants in their studies in the discussion of limitations.

Finally, we note that independence was largely framed as a goal for rehabilitation and community reintegration. However, some papers raised concerns about the Western cultural perspective of independence as an ultimate objective. Discussion of independence was more prevalent than autonomy in this body of literature and, in congruence with findings from Andrade et al. [34], these terms were sometimes conflated. For example, aspects of self-care, self-management, or cognitive capacity were referred to as autonomy that, in contrast, is more conventionally defined as the liberty to perform actions and make decisions according to a person’s values [35]. Further ethical analysis of the theoretical and pragmatic distinctions between independence and autonomy tailored to the SCI context is warranted.

### Limitations

We searched two databases and excluded abstracts and papers that were not available in English-full text. Publications by corresponding authors in Western countries may be overrepresented in this sample as a result. We recognize that the term ethics is broad and multifaceted, and that the papers included in this study do not necessarily represent the entire scope of relevant literature.

## Conclusion

The range of distinct concepts identified here reflects the breadth and impact of ethical implications in SCI. Future research on the perspectives, experiences, and values of individuals with SCI will benefit from the incorporation of an intersectional lens and more detailed attention to participant demographic variables pertaining to health outcomes.

## Supplementary information

References of extracted studies

## Data Availability

The data supporting the findings of this study, including all relevant raw data, is available from the corresponding author upon reasonable request.
