# Supplementary material for: Strengths, gaps, and future directions on the landscape of ethics-related research for spinal cord injury
Source: Spinal Cord. 2023 Apr 18;61(9):477–82. doi: 10.1038/s41393-023-00897-z (PMC10495258; doi:10.1038/s41393-023-00897-z)
Supplement: Supplementary file 1 — References of extracted studies [file 41393_2023_897_MOESM1_ESM.docx]

**Citations**

1. Anderson KD, Cowan RE, Horsewell J. Facilitators and barriers to spinal cord injury clinical trial participation: multi-national perspective of people living with spinal cord injury. J Neurotrauma. 2016; https://doi.org/10.1089/neu.2015.4064.
2. Arya S, Xue S, Embuldeniya A, Narammalage H, da Silva T, Williams S. et al. Coping strategies used by traumatic spinal cord injury patients in Sri Lanka: a focus group study. Disabil Rehabil. 2016; https://doi.org/10.3109/09638288.2015.1111433.
3. Barclay L, Lentin P, Bourke-Taylor H, McDonald R. The experiences of social and community participation of people with non-traumatic spinal cord injury. Aust Occup Ther J. 2018; https://doi.org/10.1111/1440-1630.12522.
4. Barclay L, McDonald R, Lentin P, Bourke-Taylor H. Facilitators and barriers to social and community participation following spinal cord injury. Aust Occup Ther J. 2015; https://doi.org/10.1111/1440-1630.12241.
5. Barone SH, Waters K. Coping and adaptation in adults living with spinal cord injury. J Neurosci Nurs. 2012; https://doi.org/10.1097/jnn.0b013e3182666203.
6. Beauregard L, Guindon A, Noreau L, Lefebvre H, Boucher N. Community needs of people living with spinal cord injury and their family. Top Spinal Cord Inj Rehabil. 2012; https://doi.org/10.1310/sci1802-122.
7. Bender AA, Burgess EO. Constructing recovery narratives: Experiences and expectations following spinal cord injury. Rehabil Nurs. 2019; https://doi.org/10.1097/rnj.0000000000000202.
8. Bhattarai M, Maneewat K, Sae-Sia W. Determinants of resilience among people who sustained spinal cord injury from the 2015 earthquake in Nepal. Spinal Cord. 2017; https://doi.org/10.1038/sc.2017.93
9. Bhattarai M, Maneewat K, Sae-Sia W. Psychosocial factors affecting resilience in Nepalese individuals with earthquake-related spinal cord injury: A cross-sectional study. BMC Psychiatry. 2018; https://doi.org/10.1186/s12888-018-1640-z
10. Boluki S, Dadgoo M, Dehkordi SN, Kamali M. The effect of knowledge & learning on perception and experience of independence among patients with spinal cord injury. Phys Ther. 2014; 4:20–24.
11. Braaf SC, Lennox A, Nunn A, Gabbe BJ. Experiences of hospital readmission and receiving formal carer services following spinal cord injury: a qualitative study to identify needs. Disabil Rehabil. 2017; https://doi.org/10.1080/09638288.2017.1313910.
12. Burkhart L, Kale IO, LaVela SL. Grief and loss among veterans with spinal cord injury: A qualitative study. Rehabil Nurs. 2020; https://doi.org/10.1097/rnj.0000000000000303.
13. Chuang C, Yang Y-O, Kuo LT. Finding a way to cope: a qualitative study of the experiences of persons with spinal cord injury. J Neurosci Nurs. 2015; https://doi.org/10.1097/jnn.0000000000000169.
14. Chun S, Lee Y. “I am just thankful”: The experience of gratitude following traumatic spinal cord injury. Disabil Rehabil. 2012; https://doi.org/10.3109/09638288.2012.687026.
15. deRoon-Cassini TA, de St. Aubin E, Valvano AK, Hastings J, Brasel KJ. Meaning-making appraisals relevant to adjustment for veterans with spinal cord injury. Psychol Serv. 2013; https://doi.org/10.1037/a0030963.
16. Dibb B, Ellis-Hill C, Donovan-Hall M, Burridge J, Rushton D. Exploring positive adjustment in people with spinal cord injury. J Health Psychol. 2013; https://doi.org/10.1177/1359105313483158.
17. Dorsett P, Geraghty T, Sinnott A, Acland R. Hope, coping and psychosocial adjustment after spinal cord injury. Spinal Cord Ser Cases. 2017; https://doi.org/10.1038/scsandc.2017.46.
18. Driver S, Warren AM, Reynolds M, Agtarap S, Hamilton R, Trost Z et al. Identifying predictors of resilience at inpatient and 3-month post-spinal cord injury. J Spinal Cord Med. 2014; https://doi.org/10.1179/2045772314y.0000000270.
19. Duggan C, Wilson C, DiPonio L, Trumpower B, Meade MA. Resilience and happiness after spinal cord injury: A qualitative study. Top Spinal Cord Inj Rehabil. 2016; https://doi.org/10.1310/sci2202-99.
20. Engkasan JP, Ng CJ, Low WY. Who decides? A qualitative study on the decisional roles of patients, their caregivers and doctors on the method of bladder drainage after spinal cord injury. Spinal Cord. 2014; https://doi.org/10.1038/sc.2014.199.
21. Evans RW, Bantjes J, Shackleton CL, West S, Derman W, Albertus Y et al. “I was like intoxicated with this positivity”: the politics of hope amongst participants in a trial of a novel spinal cord injury rehabilitation technology in South Africa. Disabil Rehabil Assist Technol. 2020; https://doi.org/10.1080/17483107.2020.1815086.
22. Farahani M, Khankeh HR, Hosseini M, Hosseini A, Dalvandi A, NorouziTabrizi K. Exploring facilitators of regaining autonomy in people with spinal cord injury: a qualitative study. IJNMR. 2021; https://doi.org/10.4103/ijnmr.IJNMR_25_20.
23. Geard A, Kirkevold M, Løvstad M, Schanke A-K. Exploring narratives of resilience among seven males living with spinal cord injury: a qualitative study. BMC Psychol. 2018; https://doi.org/10.1186/s40359-017-0211-2.
24. Gifre M, del Valle A, Gil À, Monreal-Bosch P. The experience of identity transformation process for people living with tetraplegia and paraplegia. Anu de Psicol. 2014; 44:361–372.
25. Goodridge D, Rogers M, Klassen L, Jeffery B, Knox K, Rohatinsky N. et al. Access to health and support services: perspectives of people living with a long-term traumatic spinal cord injury in rural and urban areas. Disabil Rehabil. 2014; https://doi.org/10.3109/09638288.2014.972593.
26. Griffiths HC, Kennedy P. Continuing with life as normal: positive psychological outcomes following spinal cord injury. Top Spinal Cord Inj Rehabil. 2012; https://doi.org/10.1310/sci1803-241.
27. Guest R, Craig A, Tran Y, Middleton J. Factors predicting resilience in people with spinal cord injury during transition from inpatient rehabilitation to the community. Spinal Cord. 2015; https://doi.org/10.1038/sc.2015.32.
28. Gupta N, Raja K. Expectations of persons with paraplegia regarding their care in India: A qualitative study. Spinal Cord Ser Cases. 2017; https://doi.org/10.1038/scsandc.2017.42.
29. Ide-Okochi A, Yamazaki Y, Tadaka E, Fujimura K, Kusunaga T. Illness experience of adults with cervical spinal cord injury in Japan: a qualitative investigation. BMC Public Health. 2013; https://doi.org/10.1186/1471-2458-13-69.
30. Jannings W, Pryor J. The experiences and needs of persons with spinal cord injury who can walk. Disabil Rehabil. 2012; https://doi.org/10.3109/09638288.2012.665126.
31. Jones KF, Dorsett P, Simpson G, Briggs L. Moving forward on the journey: spirituality and family resilience after spinal cord injury. Rehabil Psychol. 2018; https://doi.org/10.1037/rep0000229.
32. Joseph C, Wahman, K, Phillips, J, Wikmar, L.N. Client perspectives on reclaiming participation after a traumatic spinal cord injury in South Africa. Phys Ther. 2016; https://doi.org/10.2522/ptj.20150258.
33. Kaiser A, Reid D, Boschen KA. Experiences of parents with spinal cord injury. Sex Disabil. 2012; https://doi.org/10.1007/s11195-011-9238-0.
34. Kennedy P, Lude P, Elfström M, Cox A. Perceptions of gain following spinal cord injury: a qualitative analysis. Top Spinal Cord Inj Rehabil*.* 2013; <https://doi.org/10.1310/sci1903-202>.
35. Kern SB, Hunter LN, Sims AC, Berzins D, Riekena H, Andrews ML et al. Understanding the changing health care needs of individuals aging with spinal cord injury. Top Spinal Cord Inj Rehabil. 2019; https://doi.org/10.1310/sci2501-62.
36. Kirshblum SC, Botticello AL, DeSipio GB, Fichtenbaum J, Shah A, Scelza W. Breaking the news: a pilot study on patient perspectives of discussing prognosis after traumatic spinal cord injury. J Spinal Cord Med*.* 2016; https://doi.org/10.1179/2045772315y.0000000013.
37. Krause JS, Edles PA. Injury perceptions, hope for recovery, and psychological status after spinal cord injury. Rehabil Psychol. 2014; https://doi.org/10.1037/a0035778.
38. Kwon BK, Ghag A, Dvorak MF, Tetzlaff W, Illes J. Expectations of benefit and tolerance to risk of individuals with spinal cord injury regarding potential participation in clinical trials. J Neurotrauma. 2012; https://doi.org/10.1089/neu.2012.2550.
39. LaVela SL, Etingen B, Miskevics S. Factors influencing self-care behaviors in persons with spinal cord injuries and disorders. Top Spinal Cord Inj Rehabil. 2016; https://doi.org/10.1310/sci2201-27.
40. Lennon A, Bramham J, Carroll À, McElligott J, Carton S, Waldron B. et al. A qualitative exploration of how individuals reconstruct their sense of self following acquired brain injury in comparison with spinal cord injury. Brain Inj. 2013; https://doi.org/10.3109/02699052.2013.848378.
41. Lindberg J, Kreuter M, Taft C, Person L-O. Patient participation in care and rehabilitation from the perspective of patients with spinal cord injury. Spinal Cord. 2013; https://doi.org/10.1038/sc.2013.97.
42. Littooij E, Leget CJ, Stolwijk-Swüste JM, Doodeman S, Widdershoven GA, Dekker J. The importance of ‘global meaning’ for people rehabilitating from spinal cord injury. Spinal Cord. 2016; https://doi.org/10.1038/sc.2016.48.
43. Littooij E, Widdershoven GAM, Stolwijk-Swüste JM, Doodeman S, Leget CJW, Dekker J. Global meaning in people with spinal cord injury: content and changes. J Spinal Cord Med*.* 2016; https://doi.org/10.1179/2045772314y.0000000290.
44. Locatelli SM, Etingen B, Heinemann A, Neumann HDM, Miskovic A, Chen D. et al. Perceptions of shared decision making among patients with spinal cord injuries/disorders. Top Spinal Cord Inj Rehabil. 2016; https://doi.org/10.1310/sci2016-00027.
45. Lysack C, Neufeld S, Dillaway H. Do risk perceptions explain sex differences in community integration and participation after spinal cord injury? J Spinal Cord Med. 2013; https://doi.org/10.1179/2045772313y.0000000146.
46. Machida M, Irwin B, Feltz D. Resilience in competitive athletes with spinal cord injury. Qual Health Res. 2013; https://doi.org/10.1177/1049732313493673.
47. Mahooti F, Raheb G, Alipour F, Hatamizadeh N. Psychosocial challenges of social reintegration for people with spinal cord injury: a qualitative study. Spinal Cord. 2020; https://doi.org/10.1038/s41393-020-0449-z.
48. McRae J, Smith C, Emmanuel A, Beeke S. The experiences of individuals with cervical spinal cord injury and their family during post-injury care in non-specialised and specialised units in UK. BMC Health Serv Res. 2020; https://doi.org/10.21203/rs.3.rs-41115/v2.
49. Mir A, Rahnama M, Naderifar M, Badakhsh M. Living in stillness, the experience of men with spinal cord injury in the first five years after the injury- A content analysis study. J Evol Med Dent Sci. 2019; https://doi.org/10.14260/jemds/2019/701.
50. Monden KR, Philippus A, Draganich C, MacIntyre B, Charlifue S. A qualitative exploration of perceived injustice among individuals living with spinal cord injury. Rehabil Psychol. 2020; https://doi.org/10.1037/rep0000301.
51. Monden KR, Trost Z, Catalano D, Garner AN, Symcox J, Driver S et al. Resilience following spinal cord injury: a phenomenological view. Spinal Cord. 2014; https://doi.org/10.1038/sc.2013.159.
52. Munce SEP, Webster F, Fehlings MG, Straus SE, Jang E, Jaglal SB. Perceived facilitators and barriers to self-management in individuals with traumatic spinal cord injury: a qualitative descriptive study. BMC Neurol. 2014; https://doi.org/10.1186/1471-2377-14-48.
53. Munce SE, Webster F, Fehlings MG, Straus SE, Jang E, Jaglal SB. Meaning of self-management from the perspective of individuals with traumatic spinal cord injury, their caregivers, and acute care and rehabilitation managers: an opportunity for improved care delivery. BMC Neurol*.* 2016; https://doi.org/10.1186/s12883-016-0534-2.
54. Nicholls E, Lehan T, Plaza SL, Deng X, Romero, JL, Pizarro JA. et al. Factors influencing acceptance of disability in individuals with spinal cord injury in Neiva, Colombia, South America. Disabil Rehabi. 2012; https://doi.org/10.3109/09638288.2011.631684.
55. Nizeyimana E, Joseph C, Phillips J. The role of self-efficacy in community reintegration among persons with traumatic spinal cord injury in South Africa. J Spinal Cord Med. 2021; https://doi.org/10.1080/10790268.2020.1855867.
56. Noreau L, Noonan V, Cobb J, Leblond J, Dumont F. Spinal cord injury community survey: understanding the needs of Canadians with SCI. Top Spinal Cord Inj Rehabil. 2014; https://doi.org/10.1310/sci2004-265.
57. Nunnerley JL, Hay-Smith EJ, Dean SG. Leaving a spinal unit and returning to the wider community: an interpretative phenomenological analysis. Disabil Rehabil. 2012; https://doi.org/10.3109/09638288.2012.723789.
58. Pacheco Barzallo D, Oña A, Gemperli A. Unmet health care needs and inequality: A cross-country comparison of the situation of people with spinal cord injury. Health Serv Res. 2021; https://doi.org/10.1111/1475-6773.13738.
59. Piatt JA, Van Puymbroeck M, Zahl M, Rosenbluth JP, Wells MS. Examining how the perception of health can impact participation and autonomy among adults with spinal cord injury. Top Spinal Cord Inj Rehabil. 2016; https://doi.org/10.1310/sci2203-165.
60. Reinhardt JD, Ruoranen K, Graf S, Horsewell J, Leiulfsrud A, Post MWM. ‘It takes two to tango …’ revisited: A qualitative study on integration and participation of people living with spinal cord injury in Switzerland. Disabil Soc. 2013; https://doi.org/10.1080/09687599.2012.732536.
61. Rohatinsky N, Goodridge D, Rogers MR, Nickel D, Linassi G. Shifting the balance: conceptualising empowerment in individuals with spinal cord injury. Health Soc Care Community. 2016; https://doi.org/10.1111/hsc.12370.
62. Scheel-Sailer A, Post MW, Michel F, Weidmann-Hügle T. Baumann Hölzle, R. Patients’ views on their decision making during inpatient rehabilitation after newly acquired spinal cord injury-a qualitative interview-based study. Health Expect. 2017; https://doi.org/10.1111/hex.12559.
63. Suarez N, Levi R, Bullington J. Regaining health and wellbeing after traumatic spinal cord injury. J Rehabil Med. 2013; https://doi.org/10.2340/16501977-1226.
64. Tagaki M. Long-term experiences of men with spinal cord injuries in Japan: a qualitative study. Forum Qual Soc Res. 2015; https://doi.org/https://doi.org/10.17169/fqs-16.2.2148.
65. Tchajkova N, Ethans K, Smith SD. Inside the lived perspective of life after spinal cord injury: A qualitative study of the desire to live and not live, including with assisted dying. Spinal Cord. 2021; https://doi.org/10.1038/s41393-021-00619-3.
66. Trezzini B, Brach M, Post M, Gemperli A. Prevalence of and factors associated with expressed and unmet service needs reported by persons with spinal cord injury living in the community. Spinal Cord. 2019; https://doi.org/10.1038/s41393-019-0243-y.
67. Van de Velde D, Bracke P, Van Hove G, Josephsson S, Vanderstraeten G. How do men with paraplegia choose activities in the light of striving for optimal participation? A qualitative study, based on a phenomenological–hermeneutical method. Disabil Soc. 2013; https://doi.org/10.1080/09687599.2012.728795.
68. Van de Velde D, Bracke P, Van Hove G, Josephsson S, Devisch I, Vanderstraeten G. The illusion and the paradox of being autonomous, experiences from persons with spinal cord injury in their transition period from hospital to home. Disabil Rehabil. 2012; https://doi.org/10.3109/09638288.2011.608149.
69. van Diemen T, van Nes IJ, van Laake-Geelen CC, Spijkerman D, Geertzen JH, Post MW. Learning self-care skills after spinal cord injury: a qualitative study. BMC Psychol. 2021; https://doi.org/10.1186/s40359-021-00659-7.
70. Zuchetto MA, Schoeller SD, Tholl AD, Lima DK, Neves da Silva Bampi L, Ross CM. The meaning of hope for individuals with spinal cord injury in Brazil. Br J Community Nurs. 2020; https://doi.org/10.12968/bjon.2020.29.9.526.
